# The Effect of Canagliflozin on High-Density Lipoprotein Cholesterol and Angiopoietin-Like Protein 3 in Type 2 Diabetes Mellitus

**DOI:** 10.1155/2024/2431441

**Published:** 2024-03-28

**Authors:** Simo Liu, Jing Ke, Xiaotong Feng, Zongwei Wang, Xin Wang, Longyan Yang, Dong Zhao

**Affiliations:** ^1^Center for Endocrine Metabolism and Immune Diseases, Beijing Luhe Hospital, Capital Medical University, Beijing, China; ^2^Institute of Medical Genomics, Biomedical Sciences College, Shandong First Medical University, Taian, Shandong, China

## Abstract

**Background:**

Diabetes mellitus is often accompanied by dyslipidemia. Sodium-glucose cotransporter-2 (SGLT2) inhibitors, as a novel therapeutic agent for the treatment of type 2 diabetes mellitus (T2DM), have been reported to exert effects on lipid, while the results remain controversial. This study is aimed at exploring the effect of SGLT2 inhibitor canagliflozin on lipid profile.

**Methods:**

This study was a single-center, open-label, nonrandomized, prospective study. Metformin (500 mg three times per day) or canagliflozin (100 mg, once daily) was administered for 12 weeks. Fasting blood samples were collected before and 12 weeks after treatment. Serum lipid profile levels and angiopoietin-like protein 3 (ANGPTL3) were determined. In animal experiment, C57BL/6 J mice were divided into three groups including control, STZ + HFD, and STZ + HFD + canagliflozin. Lipid profile and plasma ANGPTL3 level were measured after 12 week's treatment. Moreover, the expression of ANGPTL3 was detected in the liver tissues.

**Results:**

There was a decreased trend in low-density lipoprotein cholesterol (LDL-c) and triglycerides (TG) after canagliflozin treatment, while canagliflozin significantly increased high-density lipoprotein cholesterol (HDL-c) level and decreased plasma ANGPTL3 level. In addition, the expression of ANGPTL3 in liver tissues decreased obviously in diabetic mice with canagliflozin treatment.

**Conclusions:**

Canagliflozin increases HDL-c level and suppresses ANGPTL3 expression in patients with T2DM and diabetic mice. The reduction of ANGPTL3 may contribute to the increase of HDL-c. However, the specific mechanism needs further research. This trial is registered with ChiCTR1900021231.

## 1. Background

Diabetes mellitus is often accompanied by dyslipidemia. Dyslipidemia is a common and major risk factor for atherosclerosis. Sodium-glucose cotransporter-2 (SGLT2) inhibitors, as a novel therapeutic agent for the treatment of type 2 diabetes mellitus (T2DM), have been used widely in clinical practice. SGLT2 inhibitors have exerted glucose-lower effects through inhibiting the reabsorption of glucose in the proximal tubules of the kidney. SGLT2 inhibitors have exerted their cardioprotective and renoprotective effects in various diseases, including T2DM, chronic kidney disease, and heart failure. A series of clinical trials have confirmed that SGLT2 inhibitors significantly reduced the risk of the composite of cardiovascular death and hospitalization for heart failure [1–5]. Some studies also showed the beneficial effects of SGLT2 inhibitors on reversal of left ventricular remodeling [6]. Moreover, SGLT2 inhibitors have been suggested to improve other risk factors of cardiovascular system in T2DM, such as blood pressure and uric acid [7, 8]. In addition, SGLT2 inhibitors improved insulin resistance in skeletal muscle, liver, adipose tissue, and other insulin-target organs via the central nervous system and sympathetic nervous system/parasympathetic nervous system [9].

It has been known that high-density lipoprotein cholesterol (HDL-c) is an important antiatherosclerosis component. Patients with T2DM are always accompanied by decreased HDL-c level [10]. Several clinical studies have suggested that SGLT2 inhibitor treatment increased both low-density lipoprotein cholesterol (LDL-c) and HDL-c levels [1, 11] in patients with T2DM. Angiopoietin-like protein 3 (ANGPTL3) is a member of the angiopoietin-like proteins [12]. ANGPTL3 is a glycoprotein produced by liver and has exerted its biological effects via inhibiting lipoprotein lipase and endothelial lipase. Data from genetic and clinical studies have shown that lower ANGPTL3 level is associated with lower plasma LDL-c, triglyceride (TG), and other lipoproteins [13]. Some studies have suggested that plasma ANGPTL3 was positively correlated with triglyceride levels and inversely correlated with HDL-c levels in newly diagnosed T2DM patients [14]. ANGPTL3 inhibition with evinacumab is being developed as an effective treatment to reduce TG and LDL-c [15].

Therefore, our studies are aimed at exploring the effect of SGLT2 inhibitor canagliflozin on HDL-c level and ANGPTL3 level in patients with T2DM and diabetic mice.

## 2. Methods and Materials

### 2.1. Study Subjects

All procedures performed in the study involving human participants were in accordance with the ethical standards of the institutional and/or national research committee and with the 1964 Helsinki Declaration and its later amendments or comparable ethical standards. The study was approved by the Ethics Committee of Beijing Luhe Hospital. Informed consents were obtained from all subjects. This study was a single-center, open-label, nonrandomized, prospective study. Metformin (500 mg three times per day) or canagliflozin (100 mg, once daily) was administered for 12 weeks. The study was conducted in patients with T2DM whose blood glucose was inadequately controlled after a lifestyle change. The main inclusion criteria were (1) age ≥ 18 years and ≦70 years. (2) All subjects were diagnosed with T2DM according to the 1999 WHO criteria and no hypoglycemic agent treatment before. (3) HbA1c ≥ 7.5% and ≦10.5%. The exclusion criteria were as follows: (1) acute or chronic hepatitis, (2) severe abnormal liver function, defined as liver enzyme ≥ 3 times normal value, (3) usage of lipid-lower agents, (4) severe abnormal renal function, defined as estimated glomerular filtration rate (eGFR) < 60 mL/min per 1.73 m^2^, and (5) in pregnancy or lactation.

### 2.2. Clinical and Laboratory Evaluation

Basic and anthropometric information such as age, gender, course of diabetes mellitus, and body weight were collected from all subjects. Glycated hemoglobin A1_C_ (HbA_1C_), lipid profile, uric acid, renal function, and liver function were measured in the clinical laboratory of our hospital.

### 2.3. Animal Experiments

The animal experiments were approved by the Animal Care and Use Committee of Capital University and were conducted in accordance with national and international guidelines. C57BL/6 J mice (Vital River Animal Centre, Beijing, China) at the age of 6 weeks old were used in the study. After 2 weeks' acclimatization, the mice were divided into three groups including control, STZ + HFD, and STZ + HFD + canagliflozin. Mice in control group were fed with normal diet, while other mice were treated with streptozotocin (STZ, 40 mg/kg, intraperitoneal injection) for continuously 2 weeks and then fed with a high-fat diet (HFD) for 12 weeks. Sodium carboxymethyl cellulose (CMC) or canagliflozin (30 mg/kg/per) was treated by gavage for 12 weeks. Body weight and blood glucose were measured before and after the treatment. After the sacrificed, blood sample and liver tissues were collected.

### 2.4. Measurement of ANGPTL3

The plasma ANGPTL3 levels in the patients were determined by human ANGPTL3 assay kit according to the manufacturer's instruction (IBL, USA) as described before. The level of plasma ANGPTL3 in mice was quantified by enzyme-linked immunosorbent assay (IBL, USA).

### 2.5. Immunochemistry Staining

Liver tissues were prepared as 5 *μ*m thick sections. The sections were treated with 3% hydrogen peroxide for 10 min to block endogenous peroxidase activity and incubated in buffered normal horse serum to prevent the nonspecific binding of antibodies. The sections were then incubated with an antibody against ANGPTL3 (1 : 1000) (Abcam, USA) overnight at 4°C and subjected to immunohistochemical analysis.

### 2.6. Statistical Analysis

In human research, continuous variables were presented as the mean ± standard deviation (SD), and the relative changes between baseline and posttreatment were expressed as percentage [(post‒baseline)/baseline × 100%]. To compare the mean difference before and after treatment in the canagliflozin and metformin groups, the paired *t*-test was used for normally distributed parameters, and the Wilcoxon signed rank test was used for nonnormally distributed parameters. Intergroup differences in baseline variables and the relative change were analyzed using the unpaired *t*-test (for normally distributed variables) or the Mann–Whitney *U* test (for nonnormally distributed variables). If the difference of baseline variables was significant, analysis of covariance (ANCOVA) with the baseline values as a covariate was performed to compare the significance of intergroup differences in the changes. In animal experiment, data were presented as means ± SD. Difference between groups was analyzed by one-way ANOVA followed by the post-hoc Tukey-Kramer test. *P* value <0.05 was considered statistically significant. All analyses were performed using SPSS statistics for Windows, version 27.0 (IBM Corp., Armonk, N.Y., USA).

## 3. Results

### 3.1. The Effect of Canagliflozin on Serum Lipid

The study included 75 participants, 46 male and 29 female, who were allocated to receive metformin (*n* = 30) or canagliflozin (*n* = 45) treatment. Supplementary table 1 shows the basic information and biochemical measurements of all subjects. There was no significant difference in age, gender, course of diabetes mellitus, HbA1c, total cholesterol (TC), HDL-c and LDL-c, and liver function. Subjects in canagliflozin group have higher body weight, triglycerides (TG), and diastolic pressure.

After treated with metformin or canagliflozin for 12 weeks, there were some obvious change in the subjects (Table 1). HbA1c decreased for about 1.6% in canagliflozin group vs. 1.23% in metformin group. Fasting blood glucose level decreased by 2.1 mmol/L and 1.91 mmol/L in canagliflozin and metformin groups, respectively. Aspartate aminotransferase (AST) (from 27.9 ± 14.2 to 21.9 ± 8.1 U/L) and total bilirubin (from 14.3 ± 6.4 *μ*mol/L to 11.8 ± 4.9*μ*mol/L) decreased after canagliflozin treatment as well. Canagliflozin obviously decreased uric acid level from 440.0 ± 136.4 *μ*mol/L to 371.8 ± 105.4 *μ*mol/L (*P* < 0.05), while slightly increased uric acid was seen after metformin treatment. There was no difference in terms of changes in systolic pressure, diastolic pressure, creatine, total bile acid and direct bilirubin, C-peptide, and electrolyte level.

**Figure 1:**
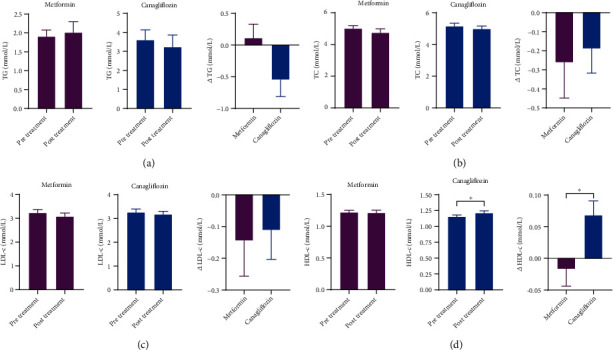
Effect of metformin and canagliflozin treatment on lipid profile. The change of lipid in patients with type 2 diabetes mellitus before and after treatment.

TC, LDL-c, and TG were unchanged in both groups, while HDL-c increased after 12 weeks' treatment with canagliflozin compared with metformin group (Figure 1).

### 3.2. The Effect of Canagliflozin on ANGPTL3 Expression

**Figure 2:**
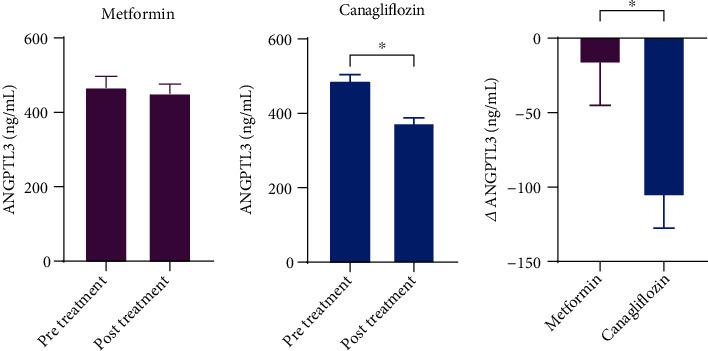
The level of plasma ANGPTL3 after metformin or canagliflozin treatment. The change of plasma ANGPTL3 level in patients with type 2 diabetes mellitus before and after treatment.

A previous study suggested that ANGPTL3 is related with HDL level and its function. Therefore, we measured ANGPTL3 level in the subjects. The results showed that canagliflozin decreased the level of plasma ANGPTL3 while metformin did not (Figure 2).

**Figure 3:**
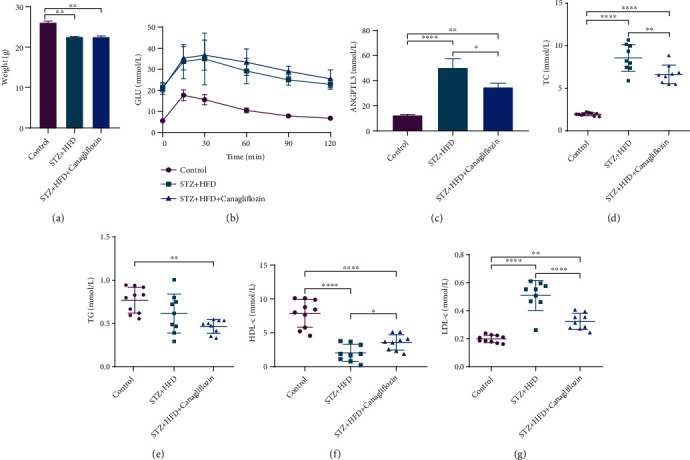
Effect of canagliflozin on glucose and lipid metabolism in diabetic mice. (a) Body weight, (b) blood glucose after intraperitoneal glucose tolerance test, (c) the level of plasma ANGPTL3, and (d–g) the level of TC, TG, HDL-c, and LDL-c.

**Figure 4:**
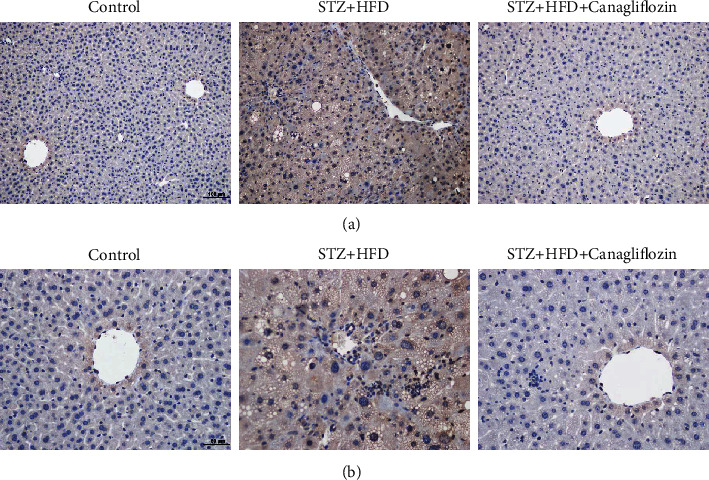
The expression of ANGPTL3 in liver tissues of diabetic mice after canagliflozin treatment. Scale bars: (a) 100 *μ*m and (b) 50 *μ*m.

To confirm the effect of canagliflozin on HDL-c level, we performed animal experiments. After 12 weeks' treatment with canagliflozin, the body weight decreased obviously compared with control and STZ + HFD groups. In the glucose tolerance test, there was no difference after canagliflozin treatment. Canagliflozin treatment significantly decreased TG, TC, and LDL-c level while increasing HDL-c level. Besides, plasma ANGPTL3 level obviously decreased after canagliflozin treatment (Figure 3). It is known that ANGPTL3 is mainly expressed in liver, so we detected ANGPTL3 expression via immunohistochemistry in the liver tissue of diabetic mice. The results showed that the expression of ANGPTL3 decreased obviously in canagliflozin group (Figure 4).

## 4. Discussion

In our study, we found that canagliflozin treatment increased HDL-c level in patients with T2DM and diabetic mice. Some studies have explored the efficacy of SGLT2 inhibitor in clinical trials in circulating lipids. A meta-analysis including studies in patients with T2DM treated with placebo or canagliflozin suggested that canagliflozin 300 mg produced the maximal increases in HDL-C [16]. Another study showed that dapagliflozin suppressed small dense LDL-c and increased HDL-2c, a favorable cardiometabolic marker [17], while there are some controversial results in LDL-c level with SGLT2 inhibitor treatment. One study showed that adding dapagliflozin 10 mg to rosuvastatin-treated diabetic patients with a well-controlled lipid profile (LDL-c after rosuvastatin: 1.5 mmol/L) did not affect plasma TC, LDL-c, ApoB, or TG levels and only modestly increased plasma HDL-c level [18]. Another study with baseline LDL-c levels < 3.1 mmol/L showed an increase of LDL-c whereas obviously reduction was seen in the subgroup with baseline LDL-c levels > 3.1 mmol/L [19]. A slight increase in LDL-c and HDL-c levels was observed also in the Canagliflozin Cardiovascular Assessment Study (CANVAS) program [20]. In our study, there was a downtrend on LDL-c level after SGLT2 inhibitor treatment for 12 weeks in human and animal experiments.

ANGPTL3 functions as an inhibitor of lipoprotein lipase (LPL) to suppress the hydrolysis of TG portion [21]. ANGPTL3 was inversely correlated with HDL-c levels in newly diagnosed T2DM patients [14]. Moreover, our previous research found the relationship between ANGPTL3 with HDL component and function in female patients with T2DM [22].

Our study showed reduced ANGPTL3 in patients with T2DM and diabetic mice after canagliflozin treatment for 12 weeks; moreover, we found the decreased expression of ANGPTL3 in the liver tissue in diabetic mice. One meta-analysis suggested that SGLT2 inhibitor can improve hepatic steatosis and liver function [23]. Another study found SGLT2 expression in the HepG2 cells, suggesting the direct effect of SGLT2 inhibitor on the liver [24]. Therefore, we guess that increased HDL-c level may be associated with the decreased ANGPTL3, while the specific mechanism needs further research. Moreover, it has been recently demonstrated that HDL function is more important than HDL-c concentration [25]. HDL-c has several functions including cholesterol efflux, anti-inflammatory, antioxidant, and antithrombotic [26]. In addition, it is thought cholesterol efflux capacity predicts cardiovascular events better than HDL-c levels [27, 28]. And apoA-I has been thought as the main cardioprotective component of HDL [29]. In the study, we have measured ANGPTL4 and aopA-I while no obvious difference was seen after canagliflozin treatment (Table 1). We did not evaluate cholesterol efflux capacity and other HDL components for the limited sample. Therefore, further studies are needed to illuminate the canagliflozin and HDL-c function and specific mechanism.

In summary, the study showed that SGLT2 inhibitor canagliflozin treatment increased HDL-c and decreased plasma ANGPTL3 level significantly. Moreover, canagliflozin decreased the expression of ANGPTL3 in the liver tissue of diabetic mice, while there are some limitations in the study. First, the sample in the study is relatively small, which may explain the nonsignificant difference in LDL-c and TG levels. Second, we excluded the use of lipid-lower agents in the clinical trials while the lifestyle was not strictly controlled.

## Figures and Tables

**Table 1:** Change of biochemical index after metformin or canagliflozin treatment.

	Metformin				Canagliflozin				*P* value^b^
	Pretreatment	Posttreatment	%change	*P* value^a^	Pretreatment	Posttreatment	%change	*P* value^a^	
Weight (kg)	73.25 ± 9.92	73.28 ± 9.90	0.04	0.363	82.4 ± 14.1	80.3 ± 13.5	-2.5	<0.001	0.233
SBP (mmHg)	129.43 ± 14.91	125.14 ± 18.58	-3.3	0.356	136.2 ± 19.1	131.1 ± 14.5	-3.7	0.138	0.902
DBP (mmHg)	79.29 ± 6.75	76.43 ± 9.9	-3.6	0.356	86.4 ± 11.0	82.5 ± 8.7	-4.5	0.116	0.837
HbA1c (%)	7.86 ± 1.58	6.63 ± 0.80	-15.6	0.001	8.5 ± 1.6	6.9 ± 0.8	-18.8	<0.001	0.319
ALT (U/L)	32.04 ± 19.05	25.43 ± 14.45	-20.6	0.045	43.0 ± 28.6	30.6 ± 17.9	-28.8	0.01	0.342
AST (U/L)	22.39 ± 8.16	21.75 ± 9.17	-2.9	0.779	27.9 ± 14.2	21.9 ± 8.1	-21.5	0.009	0.103
GGT (U/L)	51.91 ± 36.22	38.57 ± 25.07	-25.7	0.043	51.0 ± 26.8	40.7 ± 24.7	-20.2	0.001	0.574
ALP (U/L)	79.0 ± 18.23	71.57 ± 15.2	-9.4	0.02	82.0 ± 20.7	75.2 ± 22.4	-8.3	0.002	0.384
TBIL (*μ*mol/L)	12.83 ± 4.58	11.98 ± 3.83	-6.6	0.257	14.3 ± 6.4	11.8 ± 4.9	-17.5	0.014	0.227
DBIL (*μ*mol/L)	3.62 ± 1.39	3.55 ± 1.22	-1.9	0.751	3.8 ± 1.5	3.6 ± 1.1	-5.3	0.19	0.543
TBA (*μ*mol/L)	3.51 ± 2.72	3.83 ± 3.70	9.1	0.49	3.0 ± 2.0	3.3 ± 2.2	10.0	0.299	0.840
GLU (mmol/L)	9.24 ± 3.09	7.33 ± 1.95	-20.7	0.007	9.5 ± 2.5	7.4 ± 1.7	-22.1	<0.001	0.773
C-peptide (ng/mL)	3.98 ± 2.13	3.12 ± 1.72	-21.6	0.067	4.0 ± 0.8	3.7 ± 0.6	-7.5	0.129	0.357
Insulin (mU/L)	38.44 ± 72.38	25.92 ± 45.47	-32.6	0.152	18.6 ± 8.6	15.8 ± 5.5	-15.2	0.208	0.259
K (mmol/L)	4.24 ± 0.30	5.74 ± 7.86	35.4	0.323	4.27 ± 0.29	4.25 ± 0.37	-0.5	0.746	0.220
Na (mmol/L)	140.21 ± 2.35	140.25 ± 2.05	0.03	0.946	139.2 ± 1.7	139.1 ± 5.6	-0.1	0.859	0.867
CL (mmol/L)	102.84 ± 2.52	103.26 ± 1.86	0.4	0.406	101.9 ± 2.3	99.2 ± 17.4	-2.6	0.336	0.361
Ca (mmol/L)	2.35 ± 0.08	2.37 ± 0.10	0.9	0.462	2.4 ± 0.1	2.38 ± 0.12	-0.8	0.175	0.155
P (mmol/l)	1.13 ± 0.15	1.14 ± 0.13	0.9	0.744	1.12 ± 0.14	1.16 ± 0.14	3.6	0.153	0.478
Mg (mmol/L)	0.87 ± 0.07	0.84 ± 0.11	-3.4	0.448	0.86 ± 0.08	0.89 ± 0.08	3.5	0.171	0.217
Cr (*μ*mol/L)	68.11 ± 14.20	71.64 ± 16.79	5.2	0.035	73.0 ± 15.5	74.7 ± 11.4	2.3	0.249	0.409
BUN (mmol/L)	5.56 ± 1.43	5.12 ± 1.23	-7.9	0.079	5.0 ± 1.2	5.4 ± 1.3	8.0	0.048	0.042
UA (*μ*mol/L)	337.71 ± 90.02	362.86 ± 93.64	7.4	0.048	440.0 ± 136.4	371.8 ± 105.4	-15.5	<0.001	0.012
*Lipid profile*									
TG (mmol/L)	1.91 ± 0.94	2.01 ± 1.54	5.2	0.653	3.6 ± 3.5	3.3 ± 4.4	-8.3	0.662	0.079
TC (mmol/L)	5.01 ± 0.96	4.74 ± 1.31	-5.4	0.172	5.2 ± 1.2	5.0 ± 1.2	-3.8	0.151	0.740
HDL-c (mmol/L)	1.22 ± 0.24	1.20 ± 0.28	-1.6	0.577	1.1 ± 0.2	1.2 ± 0.3	9.1	0.005	0.024
LDL-c (mmol/L)	3.23 ± 0.76	3.08 ± 0.81	-4.6	0.210	3.3 ± 0.9	3.1 ± 0.9	-6.1	0.236	0.821
ANGPTL3 (ng/mL)	467.3 ± 134.7	451.0 ± 114.6	-3.5	0.579	477.9 ± 139.4	369.4 ± 109.2	-22.7	<0.001	0.015
ANGPTL4	—	—	—	—	248.4 ± 132.4	224.2 ± 97.1	-9.7	0.215	—
ApoA1	—	—	—	—	9.19 ± 4.05	9.14 ± 3.60	-0.5	0.726	—

^a^ *P* value for the intragroup comparison (pre- vs. posttreatment values in metformin or canagliflozin group). ^b^*P* value for intergroup comparison (metformin vs. canagliflozin group in the changes from pre- to posttreatment).

## Data Availability

The datasets used and/or analyzed during the current study are available from the corresponding authors on reasonable request.

## Supplementary Materials

Supplementary Materials Supplementary table 1: basic information and clinical data in metformin and canagliflozin groups.
