# Supplementary material for: The Effect of Canagliflozin on High-Density Lipoprotein Cholesterol and Angiopoietin-Like Protein 3 in Type 2 Diabetes Mellitus
Source: J Diabetes Res. 2024 Mar 28;2024:2431441. doi: 10.1155/2024/2431441 (PMC10994702; doi:10.1155/2024/2431441)
Supplement: Supplementary Materials — Supplementary table 1: basic information and clinical data in metformin and canagliflozin groups. [file 2431441.f1.docx]

**Supplementary materials**

Supplementary table 1 Basic information and clinical data in metformin and canagliflozin groups

|  | All | Metformin | Canagliflozin | *P* value |
| --- | --- | --- | --- | --- |
| N | 75 | 30 | 45 |  |
| M/F | 46/29 | 15/15 | 31/14 | 0.116 |
| Course (y) | 2（0.5, 4） | 2（0.5, 3） | 2（1.0, 5.5） | 0.095 |
| Age (year) | 48.2±13.0 | 49 ± 12.8 | 47.7 ± 13.3 | 0.666 |
| BW(kg) | 78.16±14.65 | 73.25 ± 9.92 | 82.4 ± 14.1 | 0.006 |
| SBP(mmHg) | 132.3±16.9 | 129.43 ± 14.91 | 136.2 ± 19.1 | 0.245 |
| DBP(mmHg） | 84.1±9.65 | 79.29 ± 6.75 | 86.4 ± 11.0 | 0.054 |
| HbA1c(%) | 8.19±1.58 | 7.86 ± 1.58 | 8.5 ± 1.6 | 0.137 |
| ALT(U/L) | 37.65±25.35 | 32.04 ± 19.05 | 43.0 ± 28.6 | 0.065 |
| AST(U/L) | 25.14±12.26 | 22.39 ± 8.16 | 27.9 ± 14.2 | 0.06 |
| GGT(U/L) | 49.93±30.51 | 51.91 ± 36.22 | 51.0 ± 26.8 | 0.93 |
| ALP(U/L) | 81.12±19.35 | 79.0 ± 18.23 | 82.0 ± 20.7 | 0.739 |
| TBIL(umol/L) | 13.58±5.71 | 12.83 ± 4.58 | 14.3 ± 6.4 | 0.482 |
| DBIL(umol/L) | 3.72±1.45 | 3.62 ± 1.39 | 3.8 ± 1.5 | 0.824 |
| TBA(umol/L) | 3.19±2.27 | 3.51 ± 2.72 | 3.0 ± 2.0 | 0.533 |
| GLU(mmol/L) | 9.49±2.81 | 9.24 ± 3.09 | 9.5 ± 2.5 | 0.865 |
| C-peptide(ng/mL) | 3.85±1.48 | 3.98 ± 2.13 | 4.0 ± 0.8 | 0.96 |
| Insulin(mU/L) | 26.06±46.87 | 38.44 ± 72.38 | 18.6 ± 8.6 | 0.432 |
| Cr(µmol/L) | 70.82±14.85 | 68.11 ± 14.20 | 73.0 ± 15.5 | 0.235 |
| BUN(mmol/L) | 5.23±1.32 | 5.56 ± 1.43 | 5.0 ± 1.2 | 0.048 |
| UA(µmol/L) | 397.55±128.0 | 337.71 ± 90.02 | 440.0 ± 136.4 | <0.001 |
| **Lipid profile** |  |  |  |  |
| TG(mmol/L) | 2.90±2.87 | 1.91 ± 0.94 | 3.6 ± 3.5 | 0.004 |
| TC(mmol/L) | 5.1±1.11 | 5.01 ± 0.96 | 5.2 ± 1.2 | 0.587 |
| HDL-C(mmol/L) | 1.17±0.24 | 1.22 ± 0.24 | 1.1 ± 0.2 | 0.274 |
| LDL-C(mmol/L) | 3.25±0.84 | 3.23 ± 0.76 | 3.3 ± 0.9 | 0.906 |
